# Analysis of biofilm bacterial communities under different shear stresses using size-fractionated sediment

**DOI:** 10.1038/s41598-017-01446-4

**Published:** 2017-05-02

**Authors:** Hongwei Fang, Yishan Chen, Lei Huang, Guojian He

**Affiliations:** State Key Laboratory of Hydro-science and Engineering, Department of Hydraulic Engineering, Tsinghua University, Beijing, 100084 China

## Abstract

Microorganisms are ubiquitous in aqueous environments and are crucial for biogeochemical processes, but their community structures and functions remain poorly understood. In this paper, a rotating reactor was designed to study the effects of substrata and flow conditions on sediment bacterial communities using 16S rRNA gene sequencing, assaying three groups of size-fractionated sediments and three different levels of applied shear stress. *Proteobacteria*, *Firmicutes*, and *Bacteroidetes* were the dominant phyla of the microbial communities, with more anaerobic bacteria and opportunistic pathogens being detected under static water conditions, while more aerobic bacteria were detected under dynamic water flow conditions. Most of the top 10 genera were present in all the samples; however, there were significant differences in the species abundance. *Paludibacter* and *Comamonadaceae_unclassified* were the most abundant genera under static and dynamic conditions, respectively. Under static water conditions, the medium-grained sediment had the highest microbial diversity, followed by the fine and coarse sediments. Under dynamic water flow conditions, a higher flow velocity corresponded to a greater microbial diversity. Overall, there was no significant difference in the community richness or diversity between the static and dynamic water flow conditions. This study is beneficial for further understanding the heterogeneities of microbial communities in natural aquatic ecosystems.

## Introduction

Microorganisms are important components of aquatic ecosystems and are widely distributed in aqueous environments^1^, but their community structures and functions remain poorly understood. Sediments provide excellent substrata for microorganism colonization and are more diverse than any other type of environment^2, 3^. Microorganisms on the sediment play a significant role in biogeochemical processes such as the biodegradation of organic matter and the cycling of nutrients^4, 5^, and the community structure ultimately determines their ecological and environmental functions^6–8^. In addition, the microbial community affects the biostabilization of sediment deposits through the structures of extracellular polymeric substances (EPS)^9, 10^, further affecting the aqueous environmental factors, such as sediment associated contaminant transport and habitat change^11–13^. For instance, de Brouwer *et al*.^14^. showed that the EPS secretion by *N. cf. brevissima* resulted in ordered three-dimensional matrix structures, which were effective in enhancing biostabilization. Thus, it is essential to study the microbial communities on natural sediment surfaces and the factors affecting these communities.

Substantial research has been done on sediment bacterial communities, including in both freshwater^15–20^ and marine systems^21–24^. In particular, Lozupone and Knight^1^ characterized the global patterns of bacterial diversity; Wang *et al*.^25^. compared the microbial communities in freshwater, intertidal wetlands, and marine sediments. Microbial communities are influenced by a large number of environmental factors, including pH, temperature, salinity, bioavailability of nutrients and electron acceptors, and the redox potential in the sediment^22, 26, 27^. The hypothesis “everything is everywhere, but the environment selects”^28^ describes how microorganisms with differing niche preferences are selected as the environment changes and how environmental heterogeneity plays a vital role in determining bacterial communities^29, 30^. The hydrodynamic condition is one of the most important environmental factors influencing bacterial community structure and diversity^31–34^. While aqueous chemistry (e.g., nutrients and dissolved organic carbon) is rather invariant at smaller scales in streams due to continuous mixing, the flow velocity is highly variable and controls multiple ecological processes^31^. Although some studies have focused on the effects of shear stress levels, most of these studies were done on non-sediment substrata (i.e., unglazed ceramic, stainless steel disks, polyvinyl-chloride straps, etc.), thus the influence of hydrodynamic shear stress levels on sediment bacterial diversity is still poorly understood.

A large number of reservoirs have been constructed in China during recent decades, which substantially changed the hydrological processes of natural rivers, causing profound and irreversible changes to river system functions^35, 36^. For instance, the sediment delivery ratio of the TGR was estimated to be 25.9% in 2013^37^, and more than half of the phosphorus load was intercepted by deposition in the reservoir^38^. The accumulation of sediment and nutrients at the bed surface would further stimulate bacteria attachment and biofilm growth^39, 40^. The microbial community is sensitive to changes in local environments, and comparing the number and types of bacteria in various environments would greatly aid attempts to assess the effects of environmental perturbations on community structures^41^. The heterogeneous hydrodynamic conditions and sediment sorting in the reservoir would profoundly influence the microbial diversity and result in variability and patchiness of microbial communities^42–44^. Thus, the primary objectives of this study were to investigate the effects of substrata and hydrodynamic conditions on the sediment bacterial community.

In this study, a rotating reactor was designed to analyze the biofilm bacterial communities in quiescent waters and under conditions of increased shear stress using 16S rRNA gene sequencing. Three groups of size-fractionated sediments, including 0.02–0.05, 0.05–0.1, and 0.1–0.2 mm sized sediments, and three different shear stress levels were separately used to study the effects of substrata and hydrodynamic conditions on the microbial communities. These studies are expected to provide a better understanding of the heterogeneities of microbial communities in natural river systems, which would be beneficial for further analyses of the effects of anthropogenic activities on aqueous environments.

## Results and Discussion

### Diversity indices

**Figure 1:**
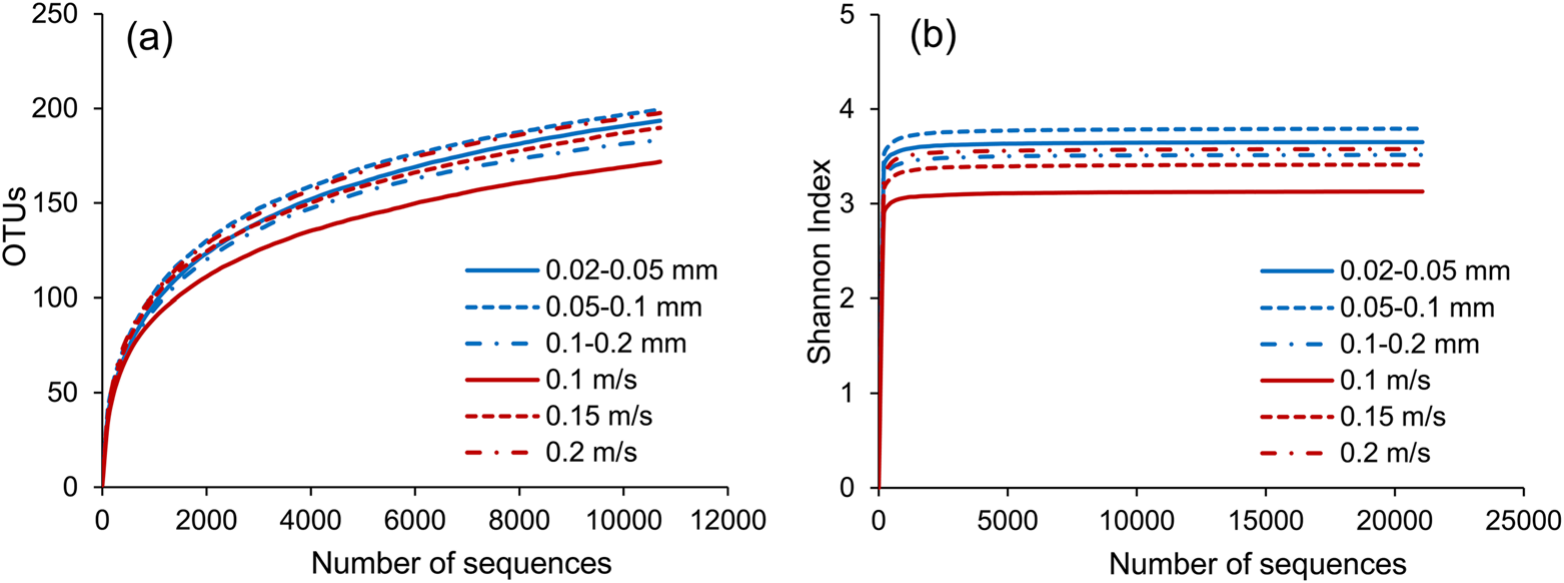
Comparisons of the *α*-diversity indices. Rarefaction curves for OTU (**a**) and Shannon index (**b**) with the sequences normalized to 30,707 reads.

A total of 197,698 16S rRNA sequences were selected for classification, with the dominant read length being approximately 395 bp. To compare the diversity indices, the sequence number of each sample was normalized to 30,707 reads, the fewest obtained among the 6 samples. The rarefaction curves for the Operational Taxonomic Unit (OTU) and Shannon index are shown in Fig. 1. It is observed that the rarefaction curves of the OTU approach a plateau after 2000 reads, at which point those of the Shannon index have plateaued. The sequencing coverage was over 0.9985, indicating that the obtained sequences reasonably represented the overall microbial communities. Under static water conditions, the medium-grained sediment had the steepest rarefaction curves with the highest richness, followed by the fine and coarse sediments. Under dynamic water flow conditions, a larger flow velocity corresponded to a greater rate increase of the rarefaction curve. Thus, the sediment exposed to a velocity of 0.2 m/s had the highest richness, followed by those exposed to velocities of 0.15 m/s and 0.1 m/s. As stated by Besemer *et al*.^31^, a larger flow velocity increased the flux of microorganisms from the bulk liquid to biofilms, thereby generating higher richness in these communities. Table 1 lists the *α*-diversity indices of the six samples, including the indices representing community richness (ACE) and those representing community diversity (Shannon and Simpson). The observed OTU and ACE index with normalized reads also supported the order of richness previously described.

**Table 1:** The OTU and *α*-diversity indices of the six samples.

Condition		OTU	ACE	Shannon	Simpson
Static	0.02–0.05 mm	196	214	3.65	0.044
	0.05–0.1 mm	198	228	3.79	0.037
	0.1–0.2 mm	182	207	3.52	0.065
Dynamic	0.1 m/s	175	206	3.13	0.122
	0.15 m/s	185	210	3.41	0.090
	0.2 m/s	194	220	3.58	0.063

Compared to the species richness (i.e., the number of species present), the diversity indices provide more information about microbial community composition, which also consider the relative abundances of different species. The Shannon index accounts for both the abundance and evenness of the species present^25^. Under static water conditions, the medium-grained sediment had a maximum Shannon diversity index value (3.79) with the highest diversity, followed by the fine (3.65) and coarse sediments (3.52). Under dynamic water flow conditions, the higher the flow velocity, the larger value of the Shannon diversity index corresponded to greater diversity. The Shannon index values were 3.13, 3.41, and 3.58 for the sediments exposed to velocities of 0.1, 0.15, and 0.2 m/s, respectively. Similar results could also be obtained for the Simpson index, a smaller value of which corresponds to a greater diversity. The grain size determined the sediment surface area available for microbial colonization and also the hydraulic conductivity^43, 44^. Fine sediment had a greater specific surface area, while the mass transfer of solutes and redox partners was limited due to the small hydraulic conductivity, which restricted microbial activity^45^. In contrast, although the coarse sediment facilitated the supply of solutes and redox partners to the sediment community, the microbial colonization was limited due to the small specific surface area. Thus, the effect of sediment size on the microbial community depended on these two interrelated factors, and there was an optimal particle size that promoted the highest diversity, i.e., the medium-grained sediment in this study. Additionally, a higher flow velocity would facilitate the supply of solutes and redox partners to the sediment community, which resulted in a greater diversity^46^. It’s worth noting that, under dynamic water flow conditions, the mass transfer at the sediment-water interface is enhanced due to the hydrodynamic forces. Thus, the microbial diversity of fine sediment will be no longer restricted by the hydraulic conductivity, and a higher microbial diversity is expected due to the greater surface area than the medium-grained and coarse sediments^44^.

**Figure 2:**
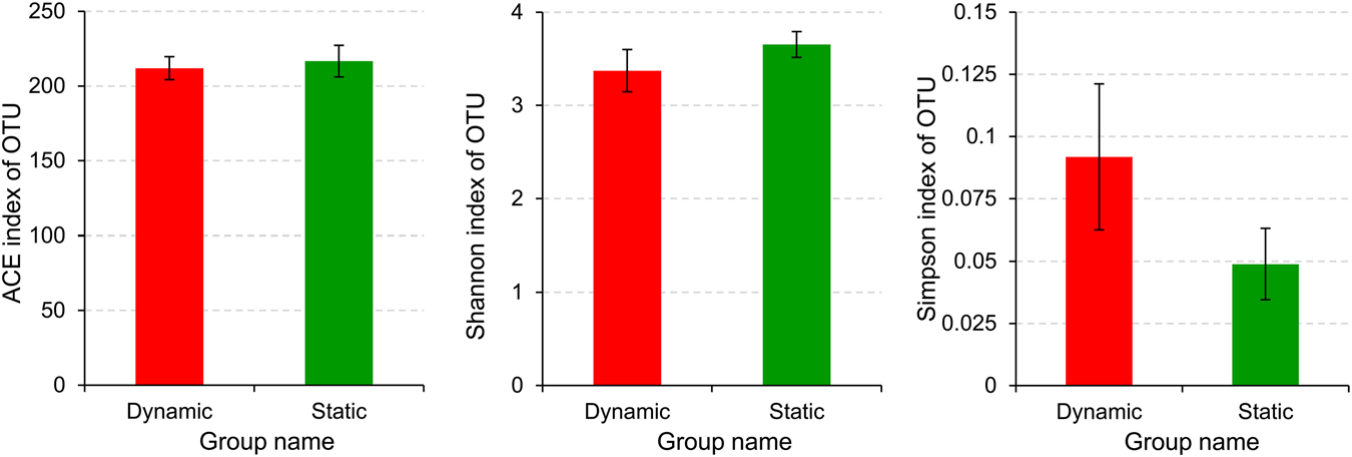
Statistical comparisons of the ACE, Shannon, and Simpson indices between static and dynamic water flow conditions, with the *p* values estimated to be 0.567, 0.141, and 0.085.

Figure 2 shows the statistical comparisons of the ACE, Shannon, and Simpson indices between static and dynamic water flow conditions. It was observed that there were slightly larger ACE and Shannon indices, and a much smaller Simpson index, under static water conditions. Rickard *et al*.^33^ and Rochex *et al*.^34^ hypothesized that lotic environments select for populations that produce stronger biofilms, consequently resulting in bacterial communities with smaller diversity. Thus, the microbial community under static water conditions had higher richness and diversity. Overall, there was no significant difference of the community richness and diversity between the static and dynamic water flow conditions, with the estimated *p* values greater than 0.05.

### Phylum-level taxonomic distribution

**Figure 3:**
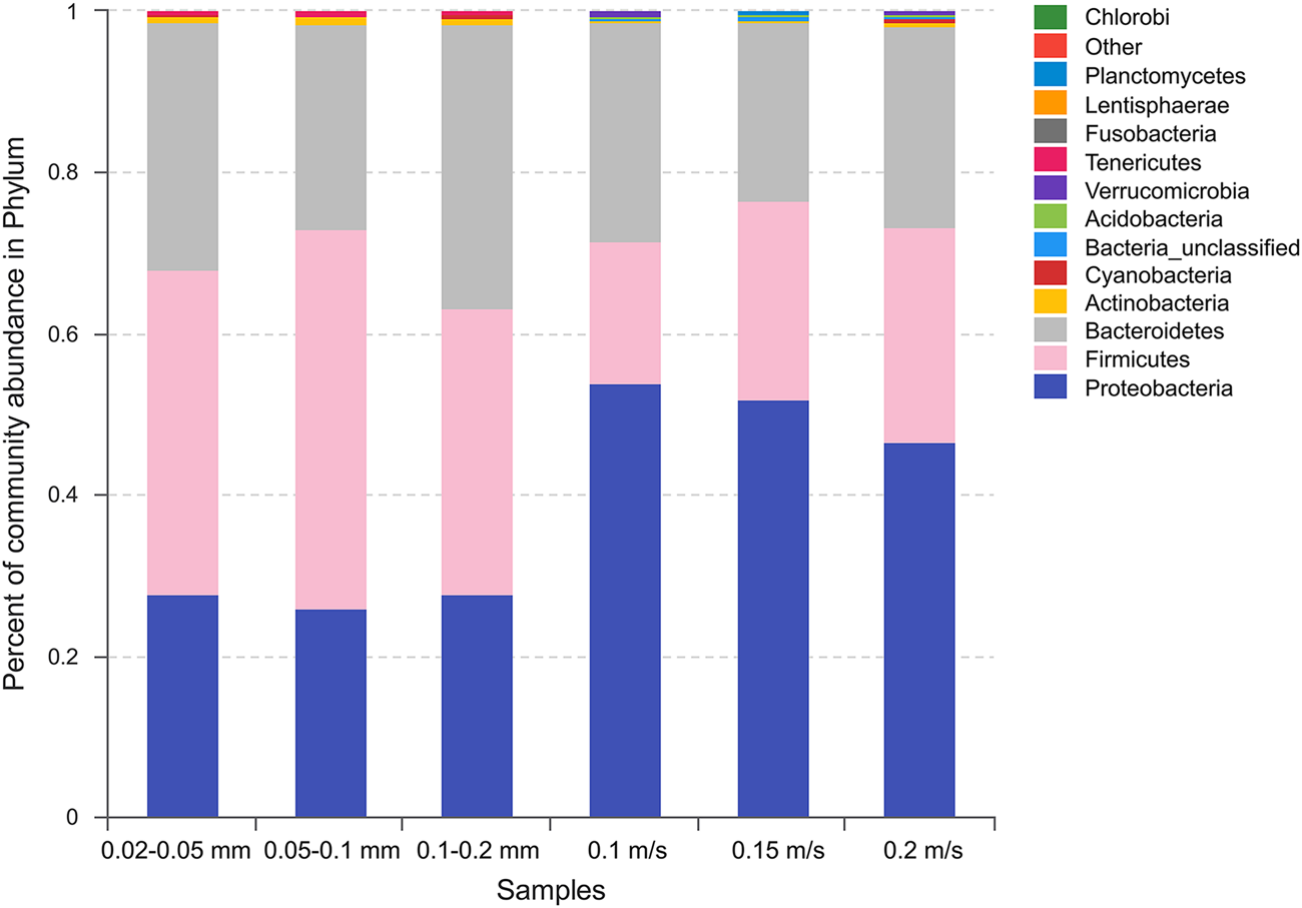
The relative abundance of the dominant bacteria at the phylum level.

The community structure of each sample was analyzed at all levels (domain, kingdom, phylum, class, order, family, genus, and species). In this paper, the phylum- and genus-level taxonomic distribution are mainly discussed. A total of 9, 10, and 11 phyla were identified in the fine, medium-grained, and coarse sediments, respectively, and 11, 14, and 13 phyla for sediments exposed to velocities of 0.1, 0.15, and 0.2 m/s, respectively. Figure 3 shows the phylum-level taxonomic distribution, showing that *Proteobacteria*, *Firmicutes*, and *Bacteroidetes* were the dominant phyla of the microbial communities, accounting for over 98% of the total abundance. The *Proteobacteria* and *Bacteroidetes* dominance was also observed in some other freshwater environments, which has been well documented^18, 47, 48^. Meanwhile, *Firmicutes*, the only phylum known to comprise endospore-formers, is the second abundant phylum represented in bacterial culture collections^49^, and the dominance of *Firmicutes* was also reported in literatures. For example, Sánchez-Andrea *et al*.^50^ concluded that *Proteobacteria*, *Firmicutes*, *Bacteroidetes*, *Acidobacteria*, and *Actinobacteria* were the 5 major phyla for the anaerobic sediments at Río Tinto River. Jiang *et al*.^18^ stated that the microbial communities of the sediment samples in Lake Chaka were dominated by sequences affiliated with *Firmicutes*, while those of the lake water samples were dominated by sequences affiliated with *Bacteroidetes*. Song *et al*.^51^ also reported the dominance of *Firmicutes* in some sediment samples of Lake Dongping, with a relative abundance of up to 40%, and a negative correlation with total phosphorus was observed for *Firmicutes*.

Although the *Proteobacteria* commonly predominates in sediment of reservoirs and lakes^52, 53^, the composition of the major *Proteobacteria* classes can differ in different environmental systems. Thus, the *Proteobacteria* classes were included in the phylum-level analysis to provide more detailed information, as listed in Table 2. It has been demonstrated that *β-Proteobacteria* usually account for a large fraction of bacteria in freshwater environments, which is negatively related to salinity, and share the ability to degrade complex organic macromolecules with *Bacteroidetes* ^17, 23^. Araya *et al*.^15^ stated that *β-Proteobacteria* might attach more easily to surfaces during initial biofilm formation and become the dominant phylogenetic group in stream water and biofilms. The phylogenetic analysis in the TGR indicated that *β-Proteobacteria* was the dominant population, ranging from 14.6 to 39.7%^16^, and an abundance of 17.9–49.0% was also observed for the surface sediment of the Yellow River^54^, which were both similar to the relative abundance of 10.5–37.2% observed in this study. In contrast, *γ-Proteobacteria* was the most significant bacteria in marine sediment, and most members of *δ-Proteobacteria* were chemoorganotrophs related to sulfate reduction, playing an important role in sulfur cycling^24^. Qu *et al*.^55^ found that *β-Proteobacteria* was the most abundant members followed by *γ-Proteobacteria* in the sediment of Guanting Reservoir.

**Table 2:** The relative abundances of the dominant phyla, including the *Proteobacteria* classes (%).

Phylum	Static condition			Dynamic condition		
	0.02–0.05 mm	0.05–0.1 mm	0.1–0.2 mm	0.1 m/s	0.15 m/s	0.2 m/s
*α- Proteobacteria*	2.3	2.4	1.1	0.5	0.5	0.7
*β- Proteobacteria*	10.8	10.5	16.8	37.2	35.9	30.2
*γ- Proteobacteria*	12.8	11.5	7.6	9.4	10.6	9.9
*δ- Proteobacteria*	1.5	1.5	2.1	6.3	4.8	5.6
*Proteobacteria*	27.6	25.9	27.7	53.8	51.9	46.6
*Firmicutes*	40.3	47.2	35.4	17.6	24.7	26.8
*Bacteroidetes*	30.8	25.4	35.3	27.3	22.1	24.9
Total	98.7	98.5	98.4	98.7	98.7	98.3

**Figure 4:**
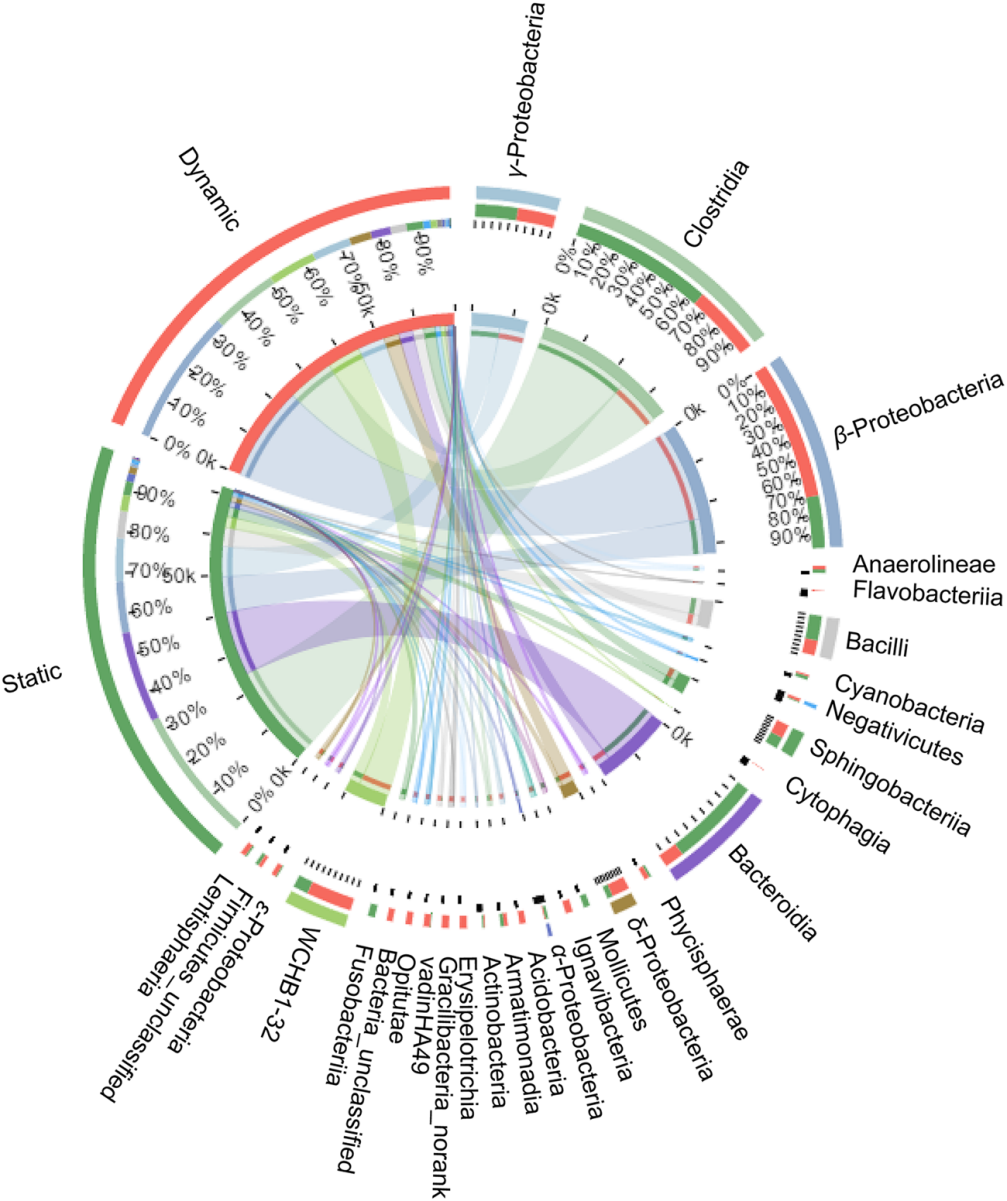
The relation between samples and the bacteria at the class level.

As shown in Fig. 3 and listed in Table 2, there are obvious differences in the relative abundances of the dominant phyla between static and dynamic water flow conditions. Overall, more *Proteobacteria* were observed under dynamic water flow conditions (46.6–53.8%), especially *β-* and *δ-Proteobacteria*. In addition, more *Firmicutes* were observed under static water conditions (35.4–47.2%), while a similar relative abundance of *Bacteroidetes* existed for these two experimental conditions with an average value of approximately 27.6%. Most species of *Firmicutes* are obligate anaerobes, whereas most species of *Proteobacteria* are strictly aerobic or facultatively anaerobic. The weak oxygen exchange under static water conditions caused a low dissolved oxygen concentration, leading to a high relative abundance of anaerobic bacteria. In contrast, more aerobic bacteria were present under dynamic water flow conditions due to the higher dissolved oxygen concentration. Figure 4 shows the relationship between samples and the bacteria at the class level. It can be observed that *Clostridia* (a *Firmicutes*, generally obligately anaerobic) preferred static water conditions as it was present at over 60%, and over 70% of *β*-*Proteobacteria* preferred dynamic water flow conditions. Moreover, *Bacteroidia* (mostly anaerobic) preferred static water conditions, while *WCHB1-32* preferred dynamic water flow conditions, both of which belong to the phylum *Bacteroidetes*. Thus, the dissolved oxygen concentration was an important factor in determining the microbial community. Bertics and Ziebis^21^ also concluded that the availability of oxidants (e.g., oxygen, nitrate, and ferric iron) played a key role in determining the presence and abundance of different taxa.

The phyla *Tenericutes* and *Fusobacteria* were only detected under static water conditions. *Tenericutes* (i.e., facultative or obligate anaerobes) are very small prokaryotes completely devoid of cell walls that tend to penetrate and grow inside the solid medium, probably affecting the biostabilization of sediment samples. *Fusobacteria* contains facultative aerobic to obligate anaerobic organisms, which are likely to occur in anoxic environments. It is worth noting that most species of these two phyla are opportunistic human pathogens.

### Genus-level taxonomic distribution

The top 10 most abundant genera under static and dynamic water flow conditions are listed in Table 3, which accounted for 61.1–72.2% of the total abundance of each sample. Under static water conditions, the medium-grained sediment appeared as a transitional community between the other two sediment conditions, sharing 9 of the top 10 genera with the fine sediment and 7 with the coarse sediment. *Paludibacter*, *Rikenellaceae_ unclassified*, *Comamonas*, *Desulfosporosinus*, *Clostridium_sensu_stricto_1*, and *Halobacteroidaceae_uncultured* were all present in these three sediment conditions. Under dynamic water flow conditions, the sediment community exposed to the medium flow velocity appeared to be a transitional community when compared to the other two flow velocities, sharing 9 of the top genera with the low flow velocity condition and 6 with the high flow velocity condition. *Comamonadaceae_unclassified*, *WCHB1-32_norank*, *Desulfocapsa*, *Aeromonas*, *Alkalibacter*, and the *Christensenellaceae_R-7_group* were present in all three flow velocities. Thus, the microbial communities of fine and medium-grained sediments had more similar microbial structures under static water conditions, and the microbial communities exposed to low and medium flow velocities were much closer to each other under dynamic water flow conditions.

**Table 3:** The 10 most abundant genera under different experimental conditions.

Rank	0.02–0.05 mm		0.05–0.1 mm		0.1–0.2 mm	
1	*Paludibacter*	0.099	*Paludibacter*	0.082	*Paludibacter*	0.192
2	*Rikenellaceae_unclassified*	0.090	*Comamonas*	0.080	*Comamonas*	0.111
3	*Comamonas*	0.083	*Rikenellaceae_unclassified*	0.070	*WCHB1-32_norank* ^†^	0.066
4	*Pseudomonas*	0.076	*Desulfosporosinus*	0.069	*Desulfosporosinus*	0.047
5	*Desulfosporosinus*	0.068	*Pseudomonas*	0.066	*Acidovorax*	0.042
6	*Gracilibacter*	0.062	*Gracilibacter*	0.066	*Halobacteroidaceae_uncultured*	0.040
7	*Clostridium_sensu_stricto_1*	0.041	*Lactococcus*	0.063	*Aeromonas*	0.040
8	*Halobacteroidaceae_uncultured*	0.041	*Halobacteroidaceae_uncultured*	0.040	*Clostridium_sensu_stricto_1*	0.034
9	*vadinBC27_wastewater-sludge_group* ^‡^	0.039	*Clostridium_sensu_stricto_1*	0.039	*Lactococcus*	0.032
10	*WCHB1-69_norank* ^*^	0.038	*WCHB1-69_norank* ^*^	0.034	*Rikenellaceae_unclassified*	0.030
Total		0.639		0.611		0.634
**Rank**	**0.1 m/s**		**0.15 m/s**		**0.2 m/s**	
1	*Comamonadaceae_unclassified*	0.303	*Comamonadaceae_unclassified*	0.261	*Comamonadaceae_unclassified*	0.193
2	*WCHB1-32_norank* ^†^	0.155	*WCHB1-32_norank* ^†^	0.115	*WCHB1-32_norank* ^†^	0.102
3	*Desulfocapsa*	0.058	*Desulfocapsa*	0.042	*WCHB1-69_norank* ^*^	0.068
4	*Aeromonas*	0.044	*Aeromonas*	0.040	*Aeromonas*	0.055
5	*Rikenellaceae_unclassified*	0.041	*Alkalibacter*	0.038	*Alkalibacter*	0.049
6	*Enterobacter*	0.030	*Enterobacter*	0.035	*Desulfocapsa*	0.049
7	*Alkalibacter*	0.025	*Rikenellaceae_unclassified*	0.032	*Zoogloea*	0.043
8	*Christensenellaceae_R-7_group*	0.025	*Rhodocyclaceae_12up*	0.031	*Desulfosporosinus*	0.043
9	*Sedimentibacter*	0.023	*Christensenellaceae_R-7_group*	0.028	*Christensenellaceae_R-7_group*	0.026
10	*Zoogloea*	0.019	*Sedimentibacter*	0.028	*Lactococcus*	0.024
Total		0.722		0.650		0.652

^‡^p_*Bacteroidetes*; c_*Bacteroidia*; o_*Bacteroidales*; f_*Rikenellaceae*; g_*vadinBC27_wastewater-sludge_group*;

^*^p_*Bacteroidetes*; c_*Sphingobacteriia*; o_*Sphingobacteriales*; f_*WCHB1-69*;

^†^p_*Bacteroidetes*; c_*WCHB1-32*.

**Figure 5:**
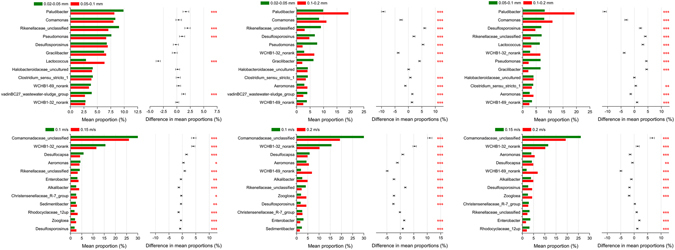
Chi-square test of the species abundance at the genus-level for sediments under different experimental conditions (0.01 < *p ≤ 0.05; 0.001 < **p ≤ 0.01; ***p ≤ 0.001).

Figure 5 shows the Chi-square test of the species abundance at the genus-level, comparing the differences of the samples under different experimental conditions. It is observed that there were significant differences in the species abundance among different samples, although most of these genera were present in all the samples. The species abundances for the fine and medium-grained sediments were relatively close to each other, with significant differences only observed for *Paludibacter*, *Rikenellaceae_unclassified*, *Pseudomonas*, *Lactococcus*, and the *vadinBC27_wastewater-sludge_group*. However, significant differences existed in the abundances of almost all the top genera for other samples, indicating the substantial effects of substrata and hydrodynamic conditions on the microbial community. In addition, the microbial communities of the fine and medium-grained sediments were more evenly distributed, while the distribution for other samples were dominated by *Paludibacter* or *Comamonadaceae_unclassified*. Under dynamic water flow conditions, the evenness increased with the increasing flow velocity, which was in agreement with the trend of the *α*-diversity results previously described.

**Figure 6:**
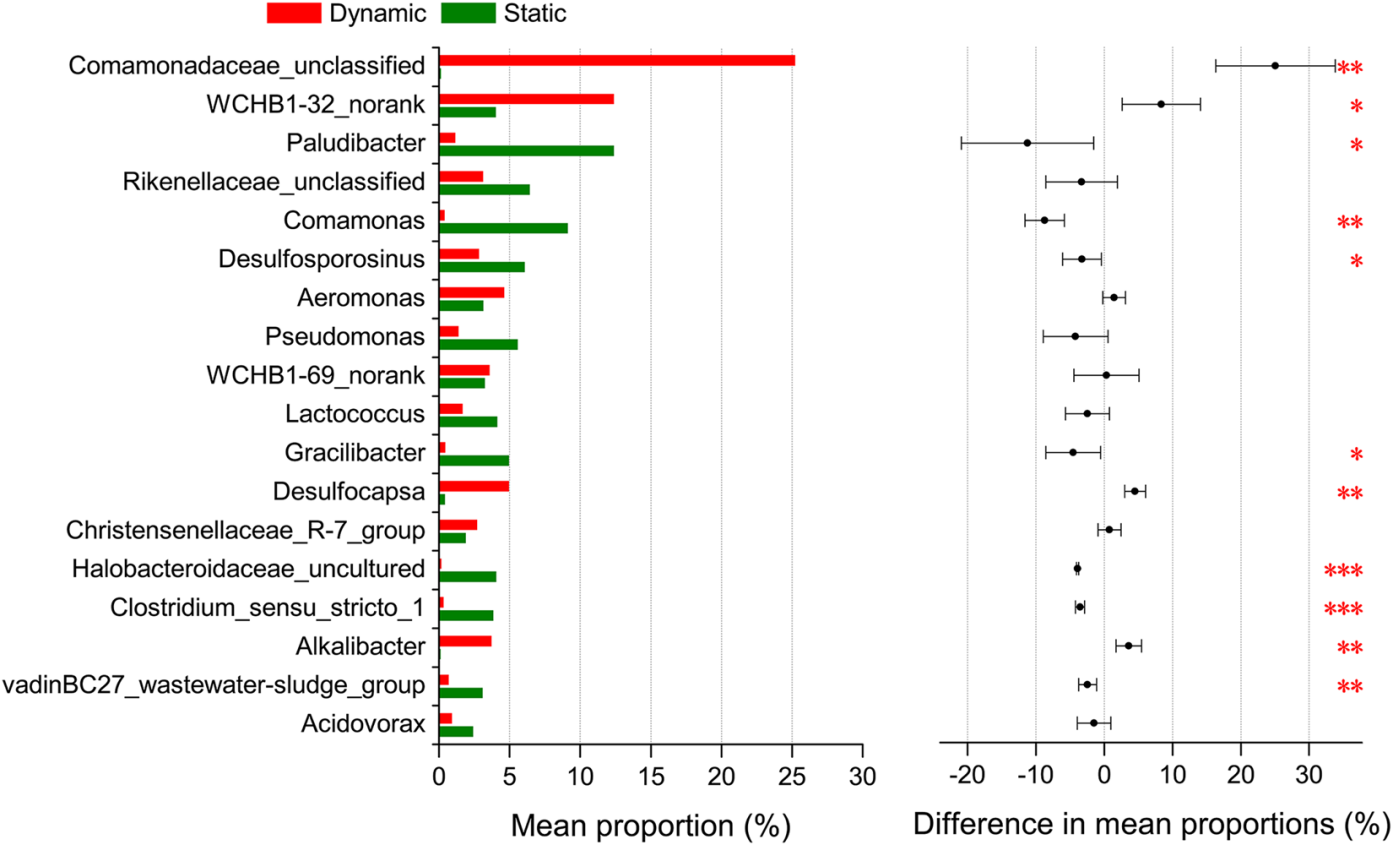
Chi-square test of the species abundance at the genus level for sediments under static and dynamic water flow conditions (0.01 < *p ≤ 0.05; 0.001 < **p ≤ 0.01; ***p ≤ 0.001).

More detailed information on the top 10 genera is listed in Table 4, including the taxonomic position and basic characteristics. The genera that were present in both the static and dynamic water flow conditions, only observed under static water conditions, or only observed under dynamic water flow conditions are separately presented. It is also observed that most species of *Firmicutes* are strictly/obligately anaerobic, whereas most species of *Proteobacteria* are aerobic or facultatively anaerobic. *Paludibacter* (within *Bacteroidetes*) was the most abundant genus under static water conditions, i.e., gram-negative, nonmotile, strictly anaerobic, chemoorganotrophic, oxidase- and catalase-negative; and *Comamonadaceae_unclassified* (within *β-Proteobacteria*) was the most abundant genus under dynamic water flow conditions, i.e., gram-negative, chemoorganotrophic or facultatively chemolithotrophic with hydrogen or carbon monoxide oxidation, possessing a strictly respiratory type of metabolism with oxygen as the terminal electron acceptor with some species also able to use nitrates, oxidase-positive. Thus, more aerobic bacteria were observed under dynamic water flow conditions affecting the corresponding microbial community, which further verified the results previously mentioned. In addition, the capability of using nitrates for *Comamonadaceae_unclassified* would lead to a more important role in the nitrogen cycling, compared to *Paludibacter*, which does not reduce nitrate. Overall, there were significant differences in the species abundance of *Paludibacter*, *Comamonas*, *Desulfosporosinus*, *Gracilibacter*, *Halobacteroidaceae_uncultured*, *Clostridum_sensu_stricto_1*, the *vadinBC27_wastewater-sludge_group*, *Comamonadaceae_unclassified*, *WCHB1-32_norank*, *Desulfocapsa*, and *Alkalibacter* for sediments under static and dynamic water flow conditions (see Fig. 6).

**Table 4:** Properties of the dominant bacteria at the genus level.

Phylum	Class	Order	Family	Genus	Characteristics^56^
**Static & Dynamic water flow conditions**					
*B*	*Bacteroidia*	*Bacteroidales*	*Rikenellaceae*	*Unclassified*	Gram-negative. Nonmotile. Anaerobic.
*B*	*WCHB1-32*	*Norank*			Phylum is a phenotypically diverse group of Gram-negative rods.
*B*	*Sphingobacteriia*	*Sphingobacteriales*	*WCHB1-69*	*Norank*	Usually nonmotile. Aerobic or facultatively anaerobic. Limited fermentative capabilities are observed in some members.
*F*	*Clostridia*	*Clostridiales*	*Peptococcaceae*	*Desulfosporosinus*	Gram-negative. Motile. Strictly anaerobic. Sulfate and thiosulfate are reduced to sulfide in the presence of lactate but not in the presence of acetate or fructose. Incomplete oxidation of organic compounds to acetate. Acetate is the fermentation end product. Autotrophic growth with hydrogen plus sulfate.
*F*	*Bacilli*	*Lactobacillales*	*Streptococcaceae*	*Lactococcus*	Gram-positive. Nonmotile. Facultatively anaerobic; catalase-negative. Chemoorganotroph. Fermentative metabolism.
*P*	*γ-Proteobacteria*	*Aeromonadales*	*Aeromonadaceae*	*Aeromonas*	Most species are motile. Facultatively anaerobic. Chemoorganotrophic, displaying oxidative and fermentative metabolism of D-glucose. Nitrate is reduced to nitrite. Usually oxidase- and catalase-positive.
**Only static water conditions**					
*B*	*Bacteroidia*	*Bacteroidales*	*Porphyromonadaceae*	*Paludibacter*	Gram-negative. Nonmotile. Strictly anaerobic. Chemoorganotrophic. Oxidase- and catalase-negative.
*B*	*Bacteroidia*	*Bacteroidales*	*Rikenellaceae*	*vadinBC27_wastewater -sludge_group*	Gram-negative. Nonmotile. Anaerobic.
*F*	*Clostridia*	*Clostridiales*	*Clostridiaceae*	*Clostridium _sensu_stricto_1*	Gram-positive. Most species are obligately anaerobic. Usually chemoorganotrophic; some species are chemoautotrophic or chemolithotrophic. Some species fix atmospheric nitrogen. Do not carry out a dissimilatory sulfate reduction. Usually catalase-negative.
*F*	*Clostridia*	*Clostridiales*	*Gracilibacteraceae*	*Gracilibacter*	Gram-positive cell-wall structure, but stains Gram-negative. Obligately anaerobic, chemoorganotrophic.
*F*	*Clostridia*	*Halanaerobiales*	*Halobacteroidaceae*	*Uncultured*	Gram-negative. Strictly anaerobic. Oxidase- and catalase-negative. Most species ferment carbohydrates. Some species may grow fermentatively on amino acids; others have a homoacetogenic metabolism or grow by anaerobic respiration while reducing nitrate, trimethylamine N-oxide, or selenate.
*P*	*β-Proteobacteria*	*Burkholderiales*	*Comamonadaceae*	*Acidovorax*	Gram-negative. Motile. Aerobic, having a strictly oxidative type of metabolism with oxygen as the terminal electron acceptor. Oxidase positive. Chemoorganotrophic.
*P*	*β-Proteobacteria*	*Burkholderiales*	*Comamonadaceae*	*Comamonas*	Gram-negative. Motile. Aerobic. Oxidase- and catalase-positive. Chemoorganotrophic, oxidative carbohydrate metabolism with oxygen as the terminal electron acceptor.
*P*	*γ-Proteobacteria*	*Pseudomonadales*	*Pseudomonadaceae*	*Pseudomonas*	Gram-negative. Motile by one or several polar flagella; rarely nonmotile. Aerobic, having a strictly respiratory type of metabolism with oxygen as the terminal electron acceptor; in some cases nitrate can be used as an alternate electron acceptor, allowing growth to occur anaerobically. Oxidase-positive or -negative. Catalase-positive. Chemoorganotrophic.
**Only dynamic water flow conditions**					
*F*	*Clostridia*	*Clostridiales*	*Eubacteriaceae*	*Alkalibacter*	Gram-positive. Nonmotile. Strictly anaerobic, catalase negative. Chemoorganoheterotroph.
*F*	*Clostridia*	*Clostridiales*	*Christensenellaceae*	*R-7_group* ^57^	Gram-negative. Anaerobic.
*F*	*Clostridia*	*Clostridiales*	*Incertae Sedis*	*Sedimentibacter*	Gram-positive or -negative. Motile. Strict anaerobe.
*P*	*β-Proteobacteria*	*Burkholderiales*	*Comamonadaceae*	*Unclassified*	Gram-negative. Chemoorganotrophic or facultatively chemolithotrophic with hydrogen or carbon monoxide oxidation. Possess a strict respiratory type of metabolism, with oxygen as the terminal electron acceptor. Some species can also use nitrates. Oxidase positive.
*P*	*β-Proteobacteria*	*Rhodocyclales*	*Rhodocyclaceae*	*12up*	Family is phenotypically, metabolically, and ecologically diverse. Includes photoheterotrophs; aerobes, anaerobes, and facultative anaerobes utilizing a number of electron acceptors; fermentative organisms; and nitrogen-fixing organisms.
*P*	*β-Proteobacteria*	*Rhodocyclales*	*Rhodocyclaceae*	*Zoogloea*	Gram-negative. Actively motile. Aerobic, having a strict respiratory type of metabolism with oxygen or nitrate as the terminal electron acceptor. Denitrification occurs with the formation of nitrogen. Oxidase-positive. Weakly catalase-positive. Chemoorganotrophic.
*P*	*γ-Proteobacteria*	*Enterobacteriales*	*Enterobacteriaceae*	*Enterobacter*	Gram-negative. Motile. Facultatively anaerobic. Glucose is fermented with the production of acid and gas (generally carbon dioxide:hydrogen = 2:1). Nitrate is reduced to nitrite. Hydrogen sulfide is not produced from thiosulfate.
*P*	*δ-Proteobacteria*	*Desulfobacterales*	*Desulfobulbaceae*	*Desulfocapsa*	Motile. Strictly anaerobic. Simple organic compounds are incompletely oxidized, with sulfate as electron donor that is reduced to sulfide. Support chemolithoautotrophic growth with carbon dioxide as carbon source. Occur in sediments of freshwater or marine habitats close to the anoxic/oxic interface.

B: Bacteroidetes; F: Firmicutes; P: Proteobacteria.

**Figure 7:**
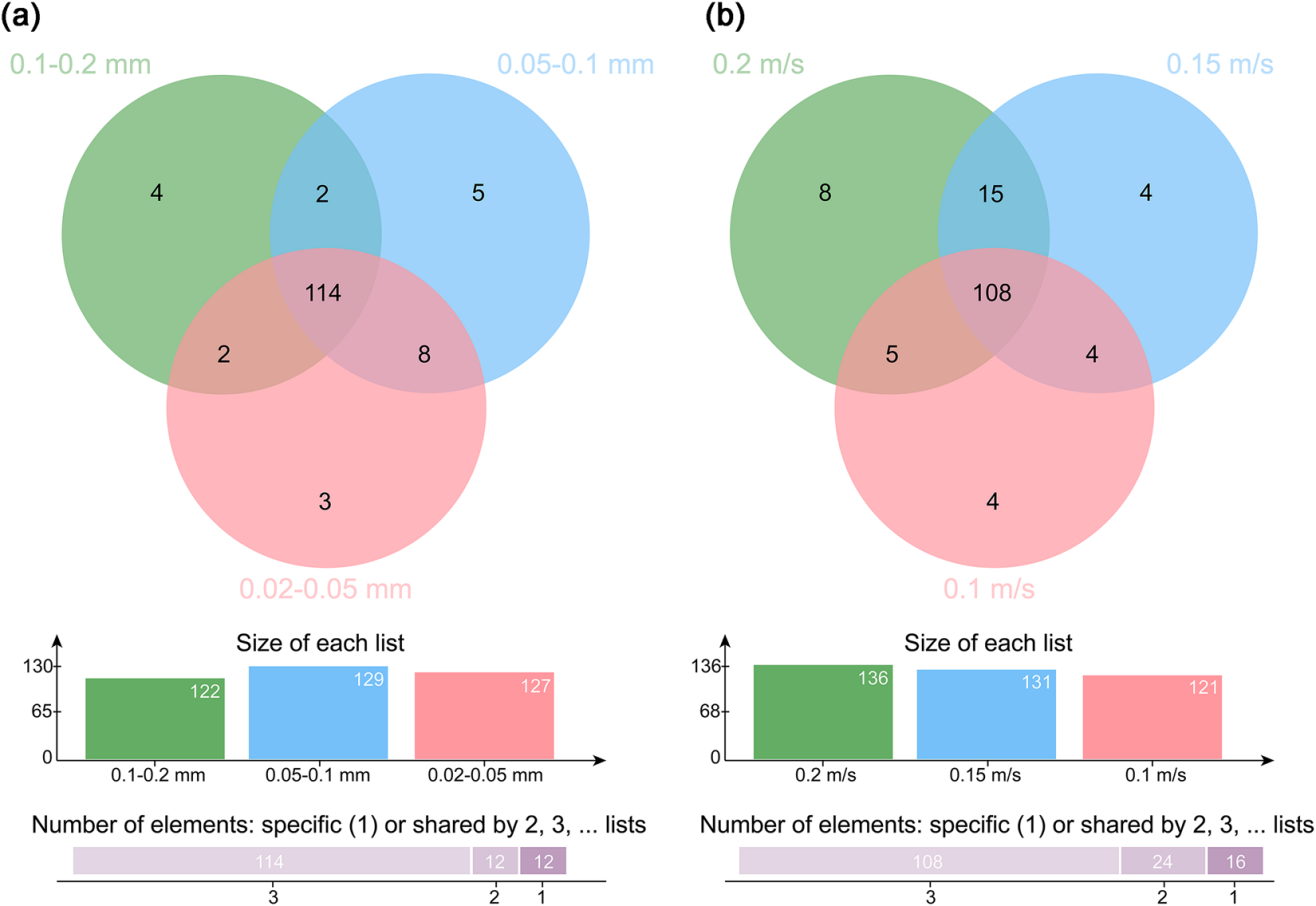
Venn diagrams of the microbial communities at the genus level under (**a**) static, and (**b**) dynamic water flow conditions.

**Figure 8:**
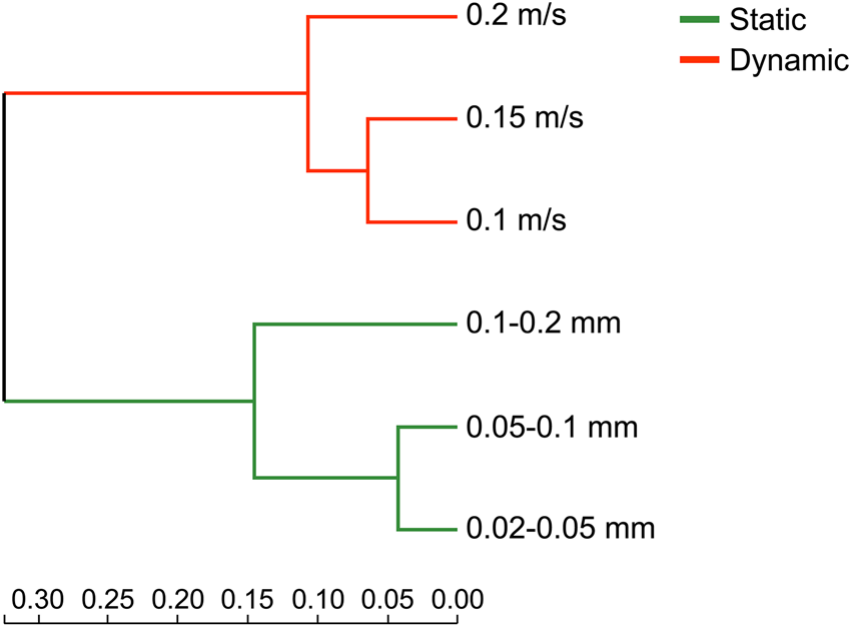
Hcluster tree of samples with the Bray-Curtis similarity index calculated using the abundance of genera.

The presence of all genera was analyzed for different sediment samples, and the Venn diagrams under static and dynamic water flow conditions are shown in Fig. 7. In total 127, 129, and 122 genera were observed for the fine, medium-grained, and coarse sediments, respectively. Among these genera, 114 were present in all three sediment samples, 12 were present in any two sediment samples, and 12 were present in only one sediment sample. Similarly, 121, 131, and 136 genera were observed for sediments exposed to flow velocities of 0.1, 0.15, and 0.2 m/s, respectively. Among these genera, 108 were present in all three sediment samples, 24 were present in any two sediment samples, and 16 were present in only one sediment sample. The clustering tree of sediment samples, based on the Bray-Curtis similarity index calculation using the genera abundances, is shown in Fig. 8. Each group of sediment samples (i.e., static and dynamic) clustered together with relatively high similarity, indicating that there were significant differences between the samples under static versus dynamic water flow conditions, and the microbial communities were selected by the hydrodynamic condition. Moreover, the medium-grained sediment community was more similar to the fine sediment community under static water conditions, and the medium flow velocity community was more similar to that of the low velocity community under the dynamic flow conditions.

### Bacterial groups with significant differences

**Figure 9:**
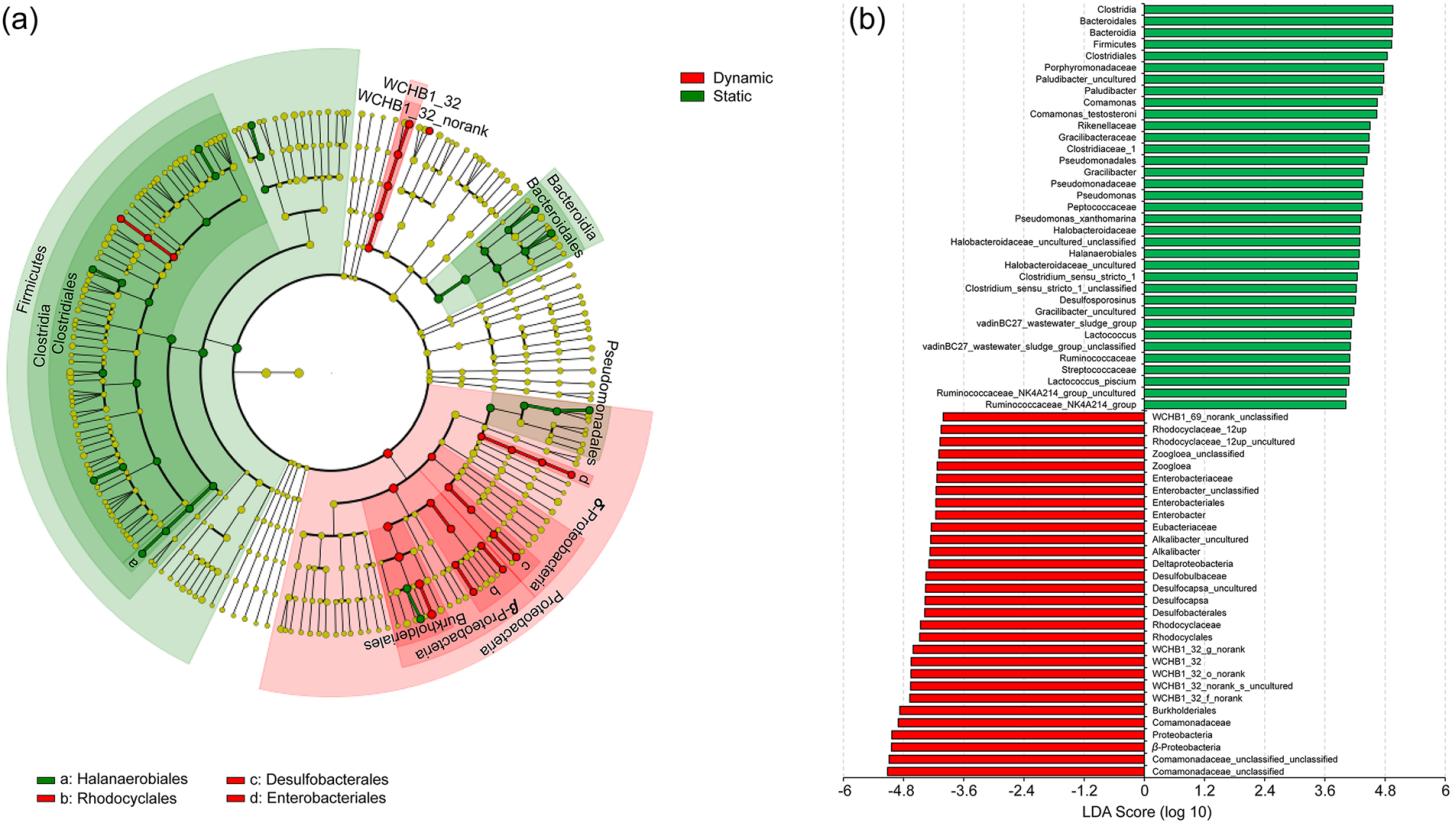
(**a**) Cladogram indicating the phylogenetic distribution of microbial lineages associated with sediments under static water conditions (green) and dynamic water flow conditions (red); and (**b**) indicator microbial groups with LDA values greater than 4.

The linear discriminant analysis (LDA) effect size (LEfSe) method was used to provide biological class explanations to establish statistical significance, biological consistency, and effect size estimation of predicted biomarkers^58^. The cladogram showing taxa with LDA values greater than 4 is presented in Fig. 9(a), and the differences are represented in the color of the most abundant bacteria, i.e., red, green, and yellow indicating dynamic or static water conditions and those that were not significant, respectively. The corresponding LDA value for each lineage is shown in Fig. 9(b). The bacterial lineages enriched under the static water conditions were *Firmicutes* (mainly of the class *Clostridia* and its orders *Clostridiales* and *Halanaerobiales* (from order to species)), *Bacteroidia*, *Pseudomonadales* (within *γ-Proteobacteria*), as shown in Fig. 9. The phylum *Proteobacteria* was enriched under dynamic water flow conditions, particularly *β-Proteobacteria* (the class and its orders *Burkholderiales* and *Rhodocydales*) and *δ-Proteobacteria* (the class and its order *Desulfobacterales*). Meanwhile, there were 3 other groups of bacteria enriched under dynamic water flow conditions, namely, *Enterobacteriales* (from order to species), *WCHB1-32* (from class to genus), and *Eubacteriaceae* (from family to species).

### Implications

In recent decades, human activities have exerted significant impacts on river systems, e.g., the hydrological regime of natural rivers have been substantially altered after dam construction. After completion of the dam, the whole reservoir generally can be classified into the river segment, transition segment and reservoir segment, which shows different hydrodynamic conditions including lotic and lentic environments. Overall, the river slope decreases and the flow slows down in the reservoir after operation, resulting in sediment deposition and sorting along the main channel. Meanwhile, the discharge of clear water causes riverbed scour downstream from the dam and sediment coarsening. Accordingly, distinct changes in the microbial community structure and diversity are expected, which would further enhance the community heterogeneities and might have different consequences in different ecosystems. For instance, more anaerobic bacteria and opportunistic pathogens probably are observed when the flow slows down, especially in a lentic environment. In addition, the microbial community exerts influences on the biostabilization of sediment deposits through effects on the biofilm structures, and the different bacteria present also play various ecological and environmental functions. Thus, the microbial community heterogeneity might significantly increase the complexities of aqueous environmental issues, and this study gives some insights into the effects of substrata and hydrodynamic conditions on the microbial community, providing references for analyzing the potential changes of microbial communities and the resultant aqueous environment effects due to anthropogenic activities.

## Conclusions

In this paper, the microbial diversity was analyzed under different shear stresses using size-fractionated sediment to study the effects of substrata and flow conditions on the microbial community. The main conclusions reached are as follows:

1. Under static water conditions, the medium-grained sediment had the highest microbial diversity, followed by the fine and coarse sediments, i.e., there was an optimal particle size with the highest microbial diversity. Under dynamic water flow conditions, a higher flow velocity corresponded to a greater microbial diversity. Overall, there was no significant difference of the microbial community richness and diversity between static and dynamic water flow conditions.
2. *Proteobacteria*, *Firmicutes*, and *Bacteroidetes* were the dominant phyla of the microbial communities, which accounted for over 98% of the total abundance. The dissolved oxygen concentration was an important factor in determining the microbial community. More anaerobic bacteria were present under static water conditions, as well as opportunistic pathogens, while more aerobic bacteria were present under dynamic water flow conditions.
3. Significant differences were detected in the species abundance among the different samples, although most of these genera were present in all the samples. *Paludibacter* and *Comamonadaceae_unclassified* were the most abundant genera under static and dynamic conditions, respectively.

## Methods

### Experimental design

**Figure 10:**
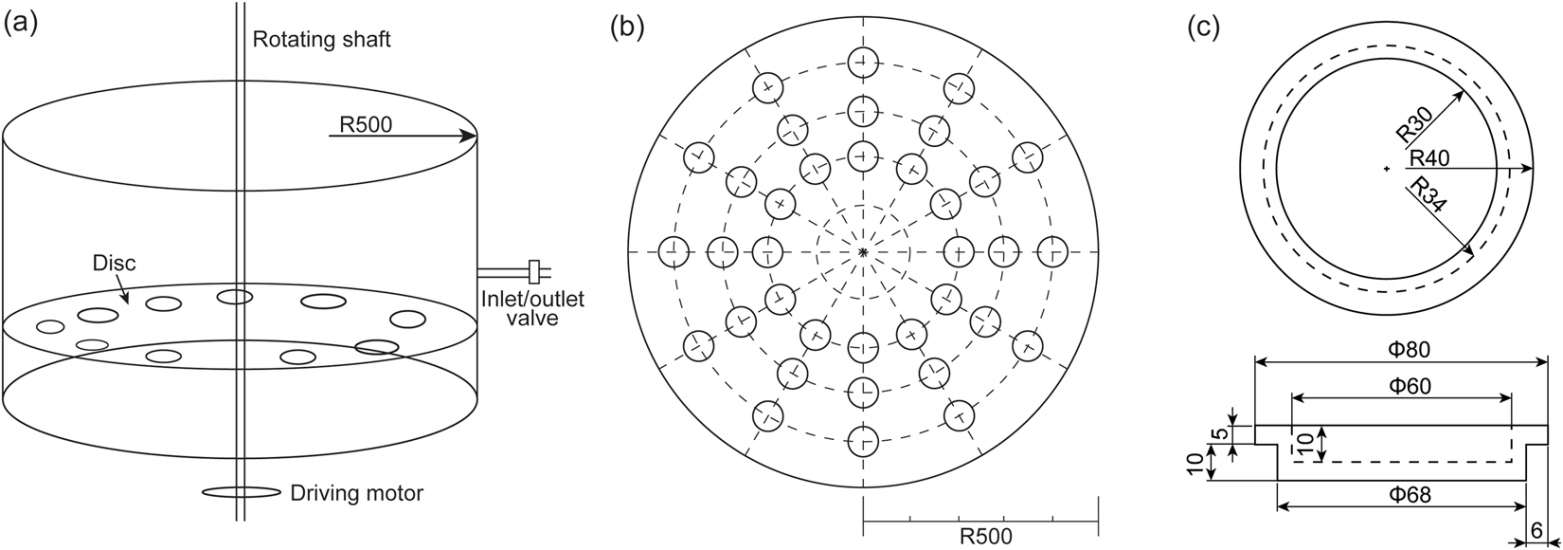
Schematic of the experimental design. (**a**) The reactor and (**b**) the rotating disc with holes arranged for placing (**c**) the sediment containers (unit: mm).

A rotating reactor with a diameter of 100 cm was designed for biofilm cultivation and microbial community analysis (see Fig. 10), primarily considering the effects of substrata and hydrodynamic conditions. In total 36 holes were arranged on the rotating disc to which the sediment containers were fixed at distances of 20, 30 or 40 cm from the center. These sediment containers had an inner diameter of 6 cm and a depth of 1 cm, with the capability of containing about 30 g sediment. As the rotational speeds are proportional to the corresponding radius, this reactor allows the simultaneous generation of different shear stresses on sediment surfaces. Moreover, the rotating reactor ensures the same water phase, i.e., the sediments are exposed to the same source community and the same aqueous chemistry condition for the different shear stresses.

Sediment used in the experiments was obtained from the Guanting Reservoir located in the northwest of Beijing, China using a columnar sampler^59^. This sediment was screened into three groups of size-fractionated sediment, i.e., fine (0.02–0.05 mm), medium-grained (0.05–0.1 mm), and coarse sediments (0.1–0.2 mm). The water from the lotus pond at Tsinghua University was used as the experimental water, having a chemical composition as follows: total nitrogen (TN) −0.60 mg/L, ammonia nitrogen (NH_3_-N) - less than 0.05 mg/L, total phosphorus (TP) - less than 0.01 mg/L, dissolved oxygen (DO) −11.68 mg/L, and chemical oxygen demand (COD_Mn_) −2.81 mg/L. Nutrients such as carbon, nitrogen, and inorganic minerals were added to the experimental water to ensure a relatively high trophic level^40^, as listed in Table 5, which was more beneficial for biofilm growth.

**Table 5:** The composition of added nutrients.

Nutrients	Glucose	KH_2_PO_4_	NaHCO_3_	MgSO_4_	NH_4_Cl	CaCl_2_
Concentration (mg/L)	500	50	1000	50	100	15

The experiments were conducted under both static and dynamic water flow conditions. The fine, medium-grained, and coarse sediments were used under the static water condition with the rotating disc immobilized. Under dynamic water conditions, the medium-grained sediment was used, and the disc was rotated at a speed of 5 rpm, i.e., rotation speeds of 0.1 m/s, 0.15 m/s, and 0.2 m/s for the sediments placed at *r* 20, 30, and 40 cm, respectively. The small rotational speed ensured a uniform annular flow in the rotating reactor, and the influences of the cross section circulation on the hydrodynamic and sediment samples can be neglected. Based on the logarithmic velocity distribution, the friction velocities U_*_ were estimated as 0.00167, 0.00255, and 0.00461 m/s, respectively, at the radius of 20, 30, and 40 cm^60^. Then the shear stresses *τ* at these three radii were estimated as 2.79 × 10^−3^ Pa, 6.50 × 10^−3^ Pa, and 2.12 × 10^−2^ Pa, respectively, which were 1–2 orders of magnitude smaller than the critical value and ensured the stability of sediment particles at the bed surface^61^. Eight samples were prepared for each experimental condition and placed at the appropriate positions before the experiment. The cylinder was then slowly filled with experimental water to a depth of 15 cm. The experiment was performed for a period of 8 weeks in the summer (25 ± 2 °C) without direct sunlight or artificial light, and the experimental water was refreshed every week through the inlet/outlet valve to replenish the nutrients. Approximately 3–5 g of surface sediment was collected weekly to determine the evolution of the biomass, and the sampling was conducted in the central part of the sediment container so that the shear stress at *r* 20, 30, and 40 cm can well represent the average condition of the shear stress exerted on these sediment samples. Previous studies have shown that the biomass achieved a peak value around day 30–42^60^. Thus, sediment samples collected at day 42 were used in the following sections for microbial community analysis.

### DNA extraction, amplification, and sequencing

Microbial DNA was extracted using the E.Z.N.A.® DNA Kit (Omega Bio-tek, Norcross, GA, US) according to the manufacturer’s protocols. The V4-V5 region of the bacterial 16S rRNA gene was amplified by Polymerase Chain Reaction (PCR) using the forward primer 515F (5′-barcode-GTGCCAGCMGCCGCGG-3′) and reverse primer 907R (5′-CCGTCAATTCMTTTRAGTTT-3′), where the barcode was an eight-base sequence unique to each sample. The following cycling parameters were used: 95 °C for 2 min, followed by 25 cycles at 95 °C for 30 s, 55 °C for 30 s, and 72 °C for 30 s and a final extension at 72 °C for 5 min. Amplicons were extracted from 2% agarose gels and purified using the AxyPrep DNA Gel Extraction Kit (Axygen Biosciences, Union City, CA, US), according to the manufacturer’s instructions, and quantified using the QuantiFluor™-ST fluorescence quantitative system (Promega, Madison, WI, US). Equimolar amounts of the purified amplicons were then pooled and paired-end sequenced (2 × 250) on an Illumina MiSeq platform according to the standard protocols.

### Processing of pyrosequencing data

Raw fastq files were demultiplexed and quality-filtered using the Quantitative Insights into Microb. Ecol. (QIIME, version 1.17). Sequences can be clustered according to similarity to each other, and here, Operational Taxonomic Units (OTUs) were clustered with a 97% similarity cutoff using UPARSE (version 7.1 http://drive5.com/uparse/). Chimeric sequences were identified and removed using UCHIME. The taxonomy of each 16S rRNA gene sequence was analyzed by the Recombination Detection Program (RDP) Classifier (http://rdp.cme.msu.edu/) against the silva (SSU115) 16S rRNA database using a confidence threshold of 70%^62^.

### Statistical analysis

The rarefaction curve and Shannon index curve (i.e., the OTU/Shannon index as a function of the sequence number) were analyzed using Mothur^63^, the slope of which indicated the degree of sequencing. Statistical analysis of *α*-diversity indices, including the ACE index for community richness and Shannon and Simpson indices for community diversity, were done using SPSS 13.0 to reflect the abundance and diversity of the microbial communities. The relationship between samples and bacteria at the class level were analyzed by Circos. The chi-square test of species abundance was also done using SPSS 13.0 to analyze the significant differences among samples. Venn diagrams of genus distributions were plotted using the VennDiagram package in the R statistical environment^64^. Cluster analysis was done using the Bray-Curtis algorithm to reflect the similarity of the measured samples. The linear discriminant analysis (LDA) effect size (LEfSe) method was also used to find indicator bacterial groups specialized within the samples of different experimental conditions^58^.
